# Identification of Small Molecules for Prevention of Lens Epithelium-Derived Cataract Using Zebrafish

**DOI:** 10.3390/cells12212540

**Published:** 2023-10-29

**Authors:** Kineret Taler, Nour Zatari, Mohammad Iqbal Lone, Shahar Rotem-Bamberger, Adi Inbal

**Affiliations:** Department of Medical Neurobiology, Institute for Medical Research—Israel-Canada, The Hebrew University-Hadassah Medical School, Jerusalem 9112002, Israel; kineret.taler@mail.huji.ac.il (K.T.); nour.zatari@mail.huji.ac.il (N.Z.); iqbalzoo84@gmail.com (M.I.L.); shahar.rotem@mail.huji.ac.il (S.R.-B.)

**Keywords:** zebrafish, *plod3*, Lysyl hydroxylase 3, cataract, posterior capsular opacification, small molecule screen, Erlotinib, 4-PBA

## Abstract

Cataract is the leading cause of blindness worldwide. It can be treated by surgery, whereby the damaged crystalline lens is replaced by a synthetic lens. Although cataract surgery is highly effective, a relatively common complication named posterior capsular opacification (PCO) leads to secondary loss of vision. PCO is caused by abnormal proliferation and migration of residual lens epithelial cells (LECs) that were not removed during the surgery, which results in interruption to the passage of light. Despite technical improvements to the surgery, this complication has not been eradicated. Efforts are being made to identify drugs that can be applied post-surgery, to inhibit PCO development. Towards the goal of identifying such drugs, we used zebrafish embryos homozygous for a mutation in *plod3* that develop a lens phenotype with characteristics of PCO. Using both biased and unbiased approaches, we identified small molecules that can block the lens phenotype of the mutants. Our findings confirm the relevance of zebrafish *plod3* mutants’ lens phenotype as a model for lens epithelium-derived cataract and add to our understanding of the molecular mechanisms that contribute to the development of this pathology. This understanding should help in the development of strategies for PCO prevention.

## 1. Introduction

Transparency of the ocular lens is critical for normal vision, and when compromised, results in cataract, one of the leading causes of blindness for many millions of people worldwide [1]. The only treatment for cataract is surgical removal of the impaired lens from its capsule, a thick basement membrane that surrounds the lens, followed by insertion of a synthetic lens into the remaining capsular bag [2]. The most common complication of this widely used procedure is posterior capsular opacification (PCO) that leads to secondary visual impairment [3]. PCO occurs when residual lens epithelial cells (LECs) that remain firmly attached to the capsule migrate to the posterior capsule where they can form two types of pathologies, fibrotic PCO and pearl PCO. In fibrotic PCO, LECs undergo an epithelial–mesenchymal transition (EMT), forming plaques of fibroblastic/myofibroblastic cells that cause lens capsule wrinkling, eventually distorting the passage of light along the visual axis [3,4]. Pearl PCO, also referred to as regenerative or regeneratory PCO, is very common and causes most vision loss post cataract surgery [5,6]. In pearl PCO, residual LECs generate globular structures named Elschnig pearls, thought to be the result of aberrant differentiation towards a lens fiber cell fate. These structures can significantly interfere with vision if they accumulate on the visual axis [7]. Both types of lens epithelial pathologies are also observed in another primary form of cataract, anterior subcapsular cataract (ASC) [3,4]. To date, the main PCO preventative strategy has been to try to eliminate all epithelial cells from the capsular bag; however, this has not proven effective with most methods, inducing other serious ocular problems [4,8,9]. An alternative approach is to devise molecular strategies to stabilize the lens epithelial cells in order to maintain the normal epithelial phenotype and prevent cataractous changes.

Most studies addressing molecular aspects of PCO development and prevention are conducted using in vitro systems, including cell culture and tissue explants [10,11]. In vivo models for PCO include transgenic (Tg) mice, genetically engineered to overexpress transforming growth factor β (TGFβ), specifically in the lens [12]. Recently, we have shown that zebrafish embryos homozygous for loss of function mutations in *plod3*, which encodes for the collagen-modifying enzyme lysyl-hydroxylase 3 (Lh3), fail to generate a normal lens capsule. This failure is likely due to the inability to properly modify Collagen IV, a major and critical component of basement membranes. Homozygous mutant larvae develop round cellular masses of partially differentiated lens fiber cells from the lens epithelium. The development of these masses resembles that of pearl PCO and ASC, and can therefore serve as an in vivo model for spontaneous development of lens epithelium-derived cataracts [13]. Importantly, human patients with reduced Lh3 function present with childhood cataracts; however, these have not been characterized [14].

In all models described above, aberrant TGFβ signaling was shown to drive the development of the lens epithelial pathologies [13,15,16,17]; however, because of its normal role in the eye, inhibition of TGFβ signaling cannot readily be used for prevention of PCO and therefore other therapeutic strategies must be identified.

In this study, we continued to use zebrafish *plod3* mutant embryos in search of molecular strategies to prevent the development of cell masses in mutant lenses, given their similarities to pearl PCO. We took two approaches to look for compounds that can inhibit development of cell masses from LECs: (1) a biased, knowledge-based approach based on the cellular abnormalities we expect from the loss of Lh3 activity and from a group of cells that exhibit increased growth; (2) an unbiased, discovery-based approach based on screening small molecule libraries. We found two small molecules that efficiently inhibit the lens phenotype, namely 4-Phenylbutyric acid (4-PBA), an ER stress and HDAC inhibitor, and Erlotinib, an epidermal growth factor receptor (EGFR) inhibitor. We further showed that the mTOR inhibitor rapamycin is not an efficient inhibitor of the pearl PCO-like phenotype. Our findings strengthen the link between the zebrafish lens phenotype and PCO in humans and contribute knowledge regarding the molecular and cellular abnormalities that promote the development of this lens epithelium-derived pathology. Hence, zebrafish *plod3* mutants can serve as an additional in vivo model in research and development of treatments for prevention of PCO.

## 2. Materials and Methods

### 2.1. Fish Lines

*plod3^vu222^* and *plod3^tv205^* have been described [13,18]. *plod3^vu222^* mutation is hypomorphic and was used for most experiments, as it better represents the situation in humans with reduced Lh3 function, given that complete loss of Lh3 function is lethal. *plod3^tv205^* embryos that are homozygous for a null mutation were used in the small molecule screen as their more severe phenotype helped in scoring rescue during the screening process. Fish were maintained under standard conditions as described in “The Zebrafish Book” [19]. 

### 2.2. Small Molecule Screen and Treatments

For the chemical screen, we used aliquots of two small molecule libraries that were purchased from Karlsruhe Institute of Technology (KIT), Germany. The first, Enzo Screen-Well FDA approved drug library V2 (BML-2843), contained almost 800 FDA-approved drugs, and the second, Enzo Screen-Well ICCB Known Bioactives library (BML-2840), contained 472 biologically active compounds.

One compound was added into each well after 1:300 dilution in a total of 300 μL egg water (“Instant Ocean” salt, 0.3 g/L reverse-osmosis-treated H_2_O) containing 0.003% N-phenylthiourea (PTU #P7629, Sigma-Aldrich, St. Louis, MO, USA). Four wells in each plate were treated with DMSO at a 1:300 dilution to serve as negative controls. Since the concentration of almost all compounds in the libraries we used was 10 mM, the final concentration of the compounds was typically 33.3 μM (except in very few cases where the original concentration was 1 mM or 5 mM). Embryos were raised at 28.5 °C and the solution of the compound was replaced with a fresh one after 24 and after 48 h. Larvae were assessed for rescue of the lens phenotype at 3 and 4 days post-fertilization (dpf). The researchers performing the screen were not informed about the identity of the compounds at the time of the screen.

The following small molecules were also used: SB-431542 (sc-204265, Santa Cruz, Dallas, TX, USA) at a final concentration of 100 μM in egg water with 0.003% PTU and 0.1% DMSO; 4-PBA (P21005, Sigma) at a final concentration of 50 µM in egg water with 0.003% PTU; Erlotinib (SML2156, Sigma) at a final concentration of 9.2 μM in egg water with 0.003% PTU and 0.2% DMSO; rapamycin (sc-3504, Santa Cruz) at a final concentration of 25 µM in egg water with 0.003% PTU and 0.1% DMSO.

In experiments for determining the time window in which a specific compound is needed, each “time frame experiment” was conducted at least twice with a total of 50 or more lenses evaluated in each experimental group.

### 2.3. Histology and Immunohistochemistry

Histology using plastic sections was performed by fixing larvae with 4% paraformaldehyde (PFA) overnight at 4 °C, subsequent washing with PBT, dehydration in EtOH series and embedding in JB4 resin (Polysciences, Inc., Warrington, PA, USA) according to the manufacturer’s instructions. We used LKB8800 Ultratome III microtome to cut 4 μm sections, which were stained with methylene blue-azure II [20]. 

We performed immunohistochemistry on cryosections. Following larvae fixation with 4% PFA overnight at 4 °C, larvae were washed with PBT, gradually transferred to 100% MeOH and kept at −20 °C. Subsequently, larvae were rehydrated with PBT and gradually transferred to 20% sucrose in 0.1 M phosphate buffer pH 7.4, incubated overnight at 4 °C and then embedded in 1.2% agarose + 5% sucrose in H_2_O. Agarose blocks were kept in 30% sucrose overnight at 4 °C and then frozen using 2-methylbutane on liquid nitrogen. We cut 16 μm sections, which were placed on Superfrost™ Plus microscope slides (Thermo Scientific, Waltham, MA, USA) and dried overnight at room temperature. Slides were washed with PBS, fixed with 4% PFA at 4 °C for 20 min, washed again with PBS, followed by 1 h blocking in 20% PHT (20% goat serum, 0.5% Triton X-100 in PBS) or 10% goat serum +2% BSA in PBT. Subsequently, sections were incubated with primary antibody overnight at 4 °C in 1% PHT (1% serum, 0.5% Triton X-100 in PBS) or 10% goat serum +2% BSA in PBT, then washed with 1% PHT or PBT for 30 min at room temperature. Incubation with secondary antibody followed, for 0.5–1.5 h at room temperature or 37 °C, and then washing with 1% PHT or PBT for 30 min and mounting in 50% glycerol or Fluoroshield Mounting Medium with DAPI (ab104139, Abcam, Cambridge, UK). 

All antibodies used in this study had been previously published and validated in zebrafish. Primary antibodies used were: rabbit anti-phospho-Smad3 (1:200) (ab52903, Abcam), rabbit anti-Phospho-p44/42 MAPK (Erk1/2) (Thr202/Tyr204) (1:200) (#9101, Cell Signaling, Danvers, MA, USA), rabbit anti-Phospho-S6 Ribosomal Protein (Ser240/244) (1:300) (Cell Signaling #2215), and monoclonal antibody 5E11 (1:200) (a kind gift from Prof. Jim Fadool, Florida State University, USA). Secondary antibodies were from Jackson ImmunoResearch: Alexa Fluor 647 AffiniPure donkey anti-rabbit IgG (H + L) (1:400), Cy3 AffiniPure donkey anti-rabbit IgG (H + L) (1:250), or Alexa Fluor 647 AffiniPure donkey anti-mouse IgG (H + L) (1:250).

### 2.4. Imaging

Imaging was performed using a Zeiss LSM 700 confocal system and Axio Imager M2 compound microscope, or with a Discovery.V8 stereoscope and AxioCam MRc digital camera (Zeiss, Oberkochen, Germany). To avoid interference by pigmentation, embryos were raised in the presence of PTU from approximately 22 hpf. For live imaging, we mounted embryos in 0.5% low-melting-point agarose (#50101, Lonza, Basel, Switzerland) in 30% Danieau’s solution (1740 mM NaCl, 21 mM KCl, 12 mM MgSO_4_·7H_2_O, 18 mM Ca(NO_3_)_2_, 150 mM HEPES buffer) and 0.01% tricaine.

### 2.5. Data Quantification and Statistical Analyses

To quantify changes in rough endoplasmic reticulum width, we used ImageJ 1.43u software [21] and measured at least 25 sites in 3–4 different cells from two different embryos for each cell type and genotype. Statistical analysis was performed using 1-tailed Student’s *t*-test.

For quantification of the effect of small molecule treatments, we classified lens position in experimental groups, relative to the position of the lens in age-matched normal larvae, as either normal, outside or inside (closer to the retina) (Figure S1). We used χ^2^ statistics to test for goodness of fit.

## 3. Results

### 3.1. Evidence for Rough Endoplasmic Reticulum Abnormalities in Lens Epithelium and Presumptive Cornea of plod3 Mutants

**Figure 1:**
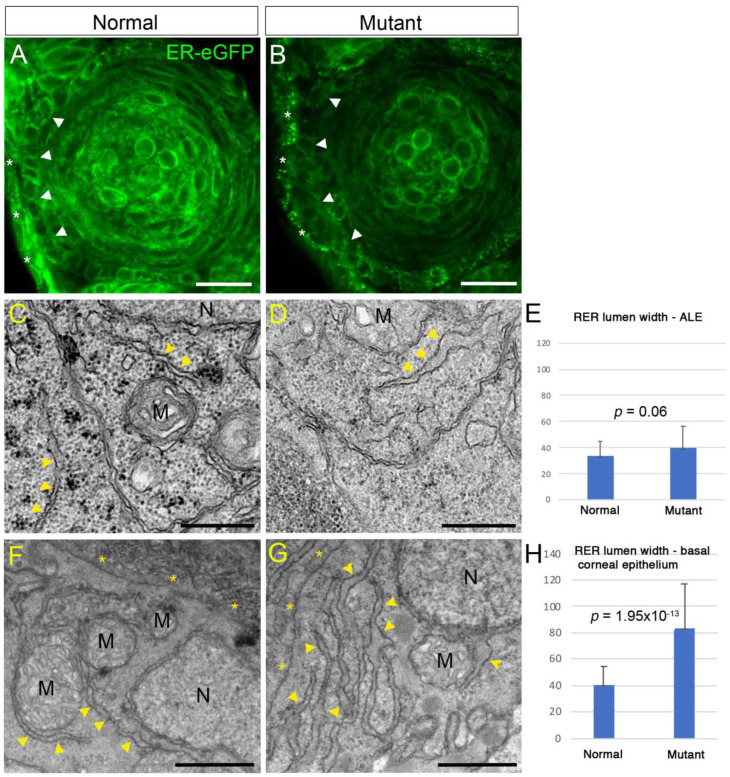
Abnormal rough endoplasmic reticulum (RER) in *plod3* mutants. (**A**,**B**) Single confocal sections from eyes of 2 dpf normal (**A**) and mutant (**B**) embryos expressing ER-eGFP. Arrowheads point at the ALE. Asterisks mark the presumptive cornea. Scale bars are 20 µm. (**C**,**D**,**F**,**G**) Transmission electron microscope images of ALE cells (**C**,**D**) and basal corneal epithelial cells (**F**,**G**) from 2 dpf normal (**C**,**F**) and mutant (**D**,**G**) embryos. Arrowheads point at RER. Asterisks in (**F**,**G**) are located by the basal cell membrane, next to the developing corneal stroma in (**F**), which is missing in (**G**). M, mitochondrion; N, nucleus. Scale bars are 500 nm (**C**,**D**) or 1 µm (**F**,**G**). (**E**,**H**) Graphs depicting measurements of RER width from mutant and normal cells in the ALE (**E**) or basal corneal epithelial cells (**H**). Numbers on the Y axis are width in µm. Error bars are standard deviation. *p* values are depicted.

Our model of lens epithelium-derived cataract in zebrafish larvae was based on a mutation that causes loss of function in the collagen-modifying enzyme Lh3. Interference with the activity of Lh3 was expected to result in accumulation of misfolded proteins, potentially leading to abnormalities in the rough endoplasmic reticulum (RER) and resulting in endoplasmic reticulum (ER) stress. Indeed, it has been suggested that mutant *Plod3* mouse embryos develop ER stress [22]. ER stress could lead to many cellular responses, including enhancing the effects of TGFβ signaling [23], the latter being a critical driver of cellular mass formation in lenses of Lh3-deficient zebrafish embryos [13]. We therefore looked for evidence of ER abnormalities in lenses of *plod3* mutant zebrafish embryos. First, we injected embryos at the one-cell stage with synthetic mRNA encoding for the ER-eGFP fusion protein that localizes to RER [24], and examined the appearance of the fluorescent signal at 2 dpf in cryosections from lenses. This time point was chosen as it is when lens capsule formation has already begun and abnormalities in the anterior lens epithelium become apparent [13,25]. The results showed a clear difference between normal and mutant embryos, whereby in normal siblings EGFP fluorescence was more abundant and mostly diffuse throughout the cytoplasm, whereas in mutants the signal was reduced and localized to puncta within the cytoplasm of lens epithelial cells and presumptive corneal cells. These puncta were particularly large in the superficial-most layer of the developing corneal epithelium (Figure 1A,B; *n* = 9 and 8 eyes, respectively). Hence, already at a relatively low resolution, the appearance of the ER was markedly different between normal and *plod3* mutant lenses and presumptive cornea cells. 

To better understand the change in RER appearance, we used transmission electron microscopy (TEM) and closely examined the anterior lens epithelium (ALE) and adjacent cells at 2 dpf. RER was much more abundant in presumptive corneal epithelial cells compared to ALE cells, in both normal siblings and in mutants. Although not abundant, some of the RER in the ALE of mutants appeared to have enlarged lumens compared to the ALE of normal siblings (Figure 1C,D; *n* = 3 eyes from each genotype). In the adjacent cells of the developing basal corneal epithelium where RER was very abundant, the lumens of RER in mutants were often mildly enlarged (Figure 1F,G). To quantify potential differences, we measured and compared the width of RER lumens in sections from normal and mutant lenses. Consistent with their appearance, lumen widths in ALE cells were on average slightly increased in mutants, although the difference was not significant (Figure 1E). In contrast, lumen widths in the basal epithelium layer of the developing cornea were significantly wider (Figure 1H). Enlarged RER lumens were likely due to accumulation of misfolded proteins and suggested the presence or development of ER stress in *plod3* mutant lens epithelium and neighboring tissues.

### 3.2. 4-PBA Rescues the Lens Phenotype of plod3^vu222^ Mutants

**Figure 2:**
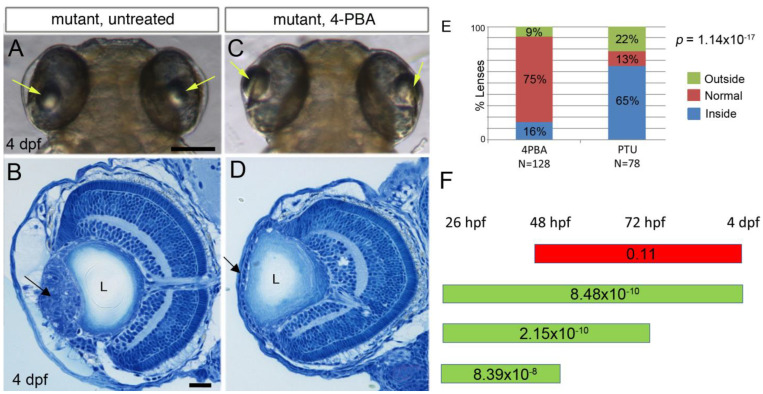
4-PBA inhibits development of lens cell masses in *plod3* mutants: (**A**,**C**) Live 4 dpf *plod3^vu222^* mutant larvae, untreated (**A**) or treated with 4-PBA from 26 hpf (**C**). Arrows point at lenses. (**B**,**D**) Histological sections of eyes from 4 dpf *plod3* mutant larvae, untreated (**B**) or treated with 4-PBA from 26 hpf (**D**). Arrow in (**B**) points at the abnormal cell mass and in (**D**) at the ALE that remains a monolayer. (**E**) Statistical analysis of the rescue with 4-PBA. Outside, normal and inside categories refer to the location of the lens relative to the normal location. N depicts the number of eyes analyzed. (**F**) Time windows of treatment with 4-PBA are represented by bars. Green bars show time windows with statistically significant rescue and red bar represents a time window in which no statistically significant rescue was observed. Numbers on bars are *p*-values. L, lens. Scale bars are 100 µm (**A**) or 20 µm (**B**).

The evidence for the presence of RER abnormalities that are consistent with ER stress prompted us to check whether inhibition of ER stress could block the formation of cellular masses in lenses of *plod3* mutants. Therefore, we treated zebrafish embryos from a cross between heterozygous *plod3^vu222^* parents with 4-Phenylbutyric acid (4-PBA), an FDA-approved drug used to treat urea cycle disorders. 4-PBA is a chemical chaperon known to alleviate ER stress [26] and also has a histone deacetylase (HDAC) inhibitor activity [27]. We treated embryos from 26 hpf until 4 dpf and assessed their lens phenotype. This treatment resulted in a significant rescue of the lens phenotype, as evidenced by localization of the lenses in live larvae (Figure 2A,C,E). Histological sections confirmed that in correctly localized lenses, the ALE remained a monolayer, without overt evidence of abnormalities (Figure 2D). The shape of the lens was abnormal, with lateral bulges, suggesting that the lens capsule was still defective (Figure 2D) (compare to a wild-type lens, Figure S2).

To better define the timing during which 4-PBA was required for inhibiting the development of lens cell masses, we repeated the treatment over several time windows: 26–48 hpf, 26–72 hpf, 26–96 hpf and 48–96 hpf. In all treatments, larvae were evaluated at 4 dpf (~96 hpf) by gross morphology. We found that when treatment was initiated at 26 hpf and continued until 48 hpf, or 72 hpf, or 96 hpf, there was a significant rescue of lens localization (Figure 2F). In contrast, when treatment was initiated from 48 hpf until 96 hpf, there was no significant difference in lens localization between treated and untreated mutants (Figure 2F). These results show that the critical time window for 4-PBA treatment is between 26 and 48 hpf, well before the formation of cell masses that begins only at 3 dpf. Hence, 4-PBA likely affects cell mass formation indirectly.

### 3.3. A Small Molecule Screen for Compounds That Can Rescue the Lens Phenotype of Lh3 Deficiency

The ability of small molecules, such as SB-431542 (an inhibitor of TGFβ type I receptor Alk5, [28]) and 4-PBA, to prevent the formation of cell masses in *plod3* mutant lenses prompted us to search for additional small molecules that can provide such rescue as potential drugs for prevention of PCO. Towards this goal, we performed a chemical screen using two small molecule libraries comprising over 1200 compounds (see Section 2).

**Figure 3:**
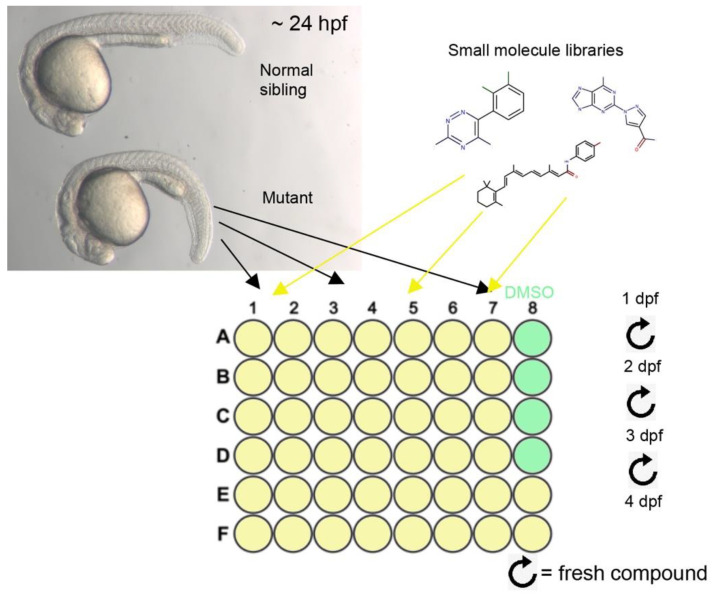
Design of the small molecule screen: Top left panel shows normal (top) and *plod3* mutant embryos at approximately 24 hpf. Mutants can be identified by their transient ventral curvature which is evident at this stage. Mutants were placed in 48-well plates, 5 per well, and compounds from the small molecule libraries were added and replaced daily until 4 dpf. Several wells in each plate were treated with DMSO as control.

*plod3* homozygous mutant embryos were identified at approximately 24 hpf based on a transient mild phenotype of abnormal ventral body curvature, and placed in 48-well plates, 5 embryos per well, without chorions (Figure 3). At 26–28 hpf, one compound was added into each well, except for four wells in each plate that received DMSO at the same dilution and served as negative controls. Compound solution was replaced with a fresh one daily, and larvae were assessed for rescue of the lens phenotype by gross morphology at 3 and 4 dpf. 

The criteria for a potentially successful “hit” were clear improvement of the localization and shape of lenses by gross morphology, and no apparent deterioration in the general health and appearance of treated embryos. For compounds that were identified as potential “hits”, we repeated the treatment twice to confirm the result. The screen yielded two compounds that provided a convincing rescue. The first was SB-431542, which we had previously shown could inhibit formation of the lens cellular masses [13]. The second compound was Erlotinib, an inhibitor of epidermal growth factor receptor (EGFR) signaling. 

### 3.4. Suppression of plod3 Mutant Lens Phenotype by Erlotinib

**Figure 4:**
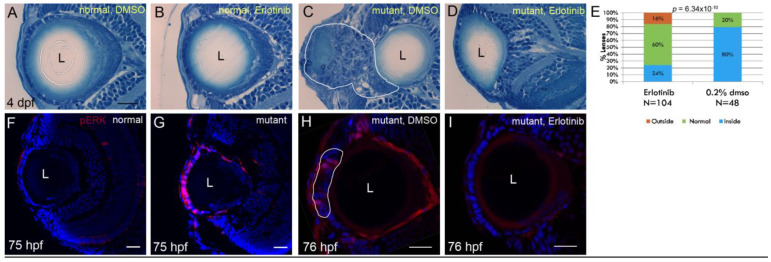
Erlotinib inhibits cell mass formation and phosphorylation of ERK1/2 in the ALE of *plod3* mutants: (**A**–**D**) Histological sections from eyes of 4 dpf normal (**A**,**B**) or *plod3* mutant (**C**,**D**) larvae. Larvae in (**A**,**C**) were treated from 1 to 4 dpf with vehicle (DMSO), whereas larvae in (**B**,**D**) were treated with Erlotinib. Scale bar is 20 µm. (**E**) Statistical analysis of the rescue of *plod3* lens phenotype by Erlotinib as determined by localization of the lens. Outside, normal and inside represent localization of the lens relative to the retina as compared to the localization in normal larvae. (**F**–**I**) Single confocal sections from eyes of 75–76 hpf normal (**F**) or *plod3* (**G**–**I**) mutant larvae, labeled with an antibody against pERK1/2 (red). Embryo in (**H**) was treated with DMSO and in (**I**) with Erlotinib. Nuclei (Dapi) are blue. L, lens. Scale bars are 20 µm.

Erlotinib binds the intracellular catalytic domain of EGFR tyrosine kinase and inhibits EGFR phosphorylation. It is used in treatment of several types of cancer as it blocks cell cycle progression by causing G1 arrest [29]. Following treatment with Erlotinib from 26 hpf until 4 dpf, development of cell masses in lenses of *plod3* mutant larvae was efficiently inhibited and lens localization was rescued (Figure 4A–E).

To better characterize EGFR activity in lenses of normal and mutant larvae, we used antibodies against phosphorylated ERK1/2 (MAPK3 and MAPK1) to label eye tissue sections. ERK1/2 are two of the main downstream proteins that are activated by phosphorylation in response to EGFR signaling [30], and hence their phosphorylation serves as an indication of EGFR activation. At 75 hpf, lens epithelium of normal larvae showed no staining for phosphorylated ERK1/2 (pERK1/2) by immunohistochemistry (Figure 4F, *n* = 10). In contrast, *plod3^vu222^* mutant lenses showed intense staining in the ALE, even though cell masses were not apparent yet (Figure 4G, *n* = 13). To confirm that Erlotinib acted by inhibiting EGFR signaling in the lens epithelium, we examined whether ERK1/2 phosphorylation was affected by the treatment. Consistent with this notion, in mutants that were treated with Erlotinib from 1 dpf, no pERK staining was present in the ALE (Figure 4I, *n* = 13), whereas in the vehicle-treated mutants, pERK-positive cells were present in developing masses (Figure 4H, *n* = 12). These results confirmed that the phosphorylation of ERK1/2 in lenses of *plod3*-deficient larvae was dependent on EGFR signaling. 

### 3.5. TGFβ Signaling Functions Upstream of EGFR Signaling in Cell Mass Formation

Inhibition of TGFβ- or EGFR-signaling prevented formation of cell masses and maintained the ALE as a monolayer, raising the possibility that these pathways interact in this pathogenic process. To test this possibility, we first defined the time windows during which the activity of these pathways was required. 

**Figure 5:**
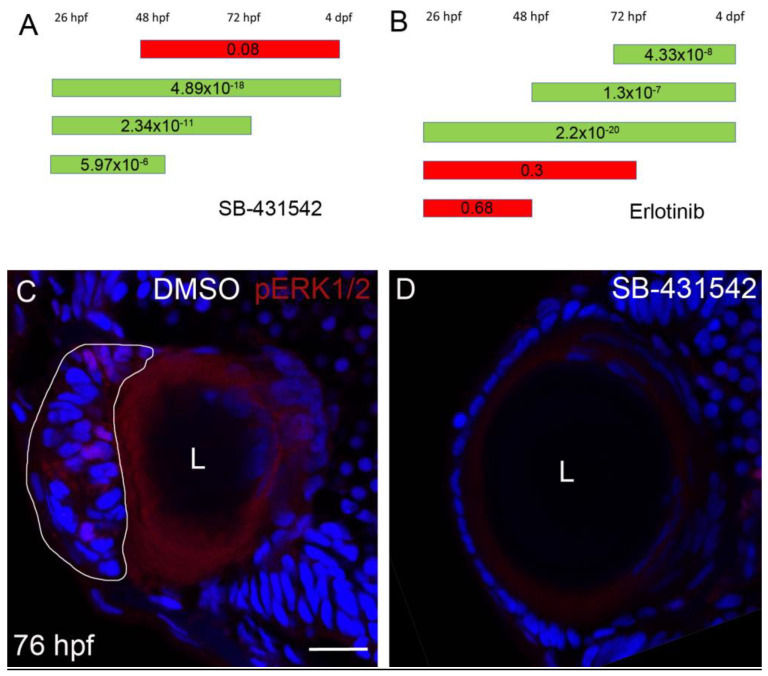
TGFβ signaling is upstream of EGFR signaling in *plod3* mutant lenses. (**A**,**B**) Timing experiments of rescue by TGFβ- and EGFR-signaling inhibition, respectively. Green bars show time windows with statistically significant rescue and red bars represent time windows in which no statistically significant rescue was observed. Numbers on bars are *p*-values. (**C**,**D**) Confocal single-plane sections from eyes of 76 hpf *plod3^vu222^* mutants treated with vehicle (**C**) or with SB-431542 (**D**) from ~26 hpf to 76 hpf and labeled for pERK1/2 (red) and nuclei (Dapi, blue). L, lens. Scale bar is 20 µm.

We had previously shown that to achieve rescue by inhibiting TGFβ signaling, the inhibition needs to be initiated by ~30 hpf [13]. To better define when TGFβ signaling promotes cell mass formation, we inhibited this pathway using SB-431542 during several time windows and assessed the phenotype by observing lens localization at 4 dpf. The time windows of the treatments were 26 hpf–48 hpf, 26 hpf–72 hpf and 26 hpf–4 dpf. When treatment was ended before 4 dpf, the solution in which embryos were raised was replaced several times to wash out the inhibitor and the embryos were kept in egg water until 4 dpf. Treatment during all these time windows resulted in statistically significant rescue, with significance increasing in the more prolonged treatments (Figure 5A). These results suggest that the critical timing for TGFβ signaling is between 30 hpf to 48 hpf, since beginning treatment at 48 hpf failed to produce rescue of the lens phenotype [13]. Interestingly, we could not detect ectopic pSmad3 signal at 48 hpf in the ALE suggesting that another arm of the TGFβ pathway was activated at this developmental stage, i.e., the non-canonical TGFβ pathway.

Next, we similarly determined the time window during which EGFR signaling is required. The initial time windows of the treatments were 26 hpf–48 hpf, 26 hpf–72 hpf and 26 hpf–4 dpf. We found that when Erlotinib was added from 26 hpf to 48 hpf or 72 hpf, no significant rescue of lens localization was achieved. To further refine the time windows in which Erlotinib acts, we added treatments from 48 or 72 hpf. Indeed, when Erlotinib was added from 26 hpf, 48 hpf or 72 hpf to 96 hpf, a significant rescue of lens localization was evident (Figure 5B). These results show that inhibition of EGFR signaling is required from 3 dpf, just prior to cell mass formation. Consistent with this conclusion, at 48 hpf, no pERK labeling was evident in the ALE of mutant or normal embryos.

Given that TGFβ signaling was required at an earlier time window, we hypothesized that it functioned upstream of EGFR signaling. To further test this hypothesis, we treated *plod3^vu222^* mutants with SB-431542 from ~30 hpf and examined pERK expression at 76 hpf. Larvae that were treated with the TGFβ receptor inhibitor showed no staining for pERK in the ALE (Figure 5C,D, *n* = 13 and 12, respectively), supporting the notion that TGFβ signaling acts upstream of, and is needed for, EGFR pathway activation. 

### 3.6. 4-PBA Inhibits Only Late TGFβ Signaling and Does Not Inhibit EGFR Signaling

Having defined the time windows during which TGFβ and EGFR signaling promoted lens cell mass formation, we wished to find whether and how the activity of these pathways was influenced by 4-PBA treatment. Therefore, we treated mutant embryos with 4-PBA and examined the effect on TGFβ and EGFR signaling pathways by examining pSmad3 and pERK1/2 labeling, respectively.

**Figure 6:**
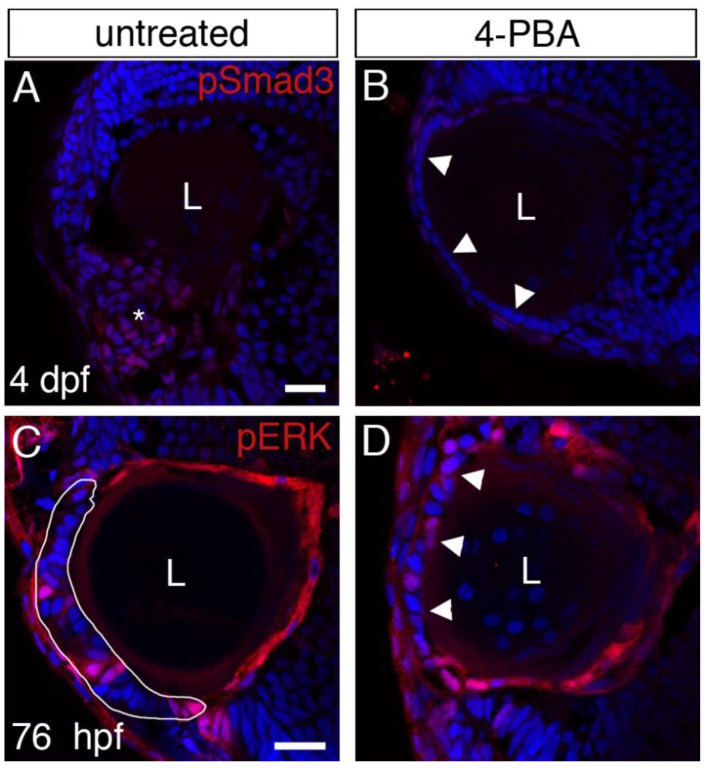
Effects of 4-PBA treatment on TGFβ and EGFR signaling. (**A**–**D**) Confocal single-plane sections from eyes of *plod3^vu222^* mutants untreated (**A**,**C**) or treated with 4-PBA (**B**,**D**) from ~26 hpf. In (**A**,**B**), treatment was until ~96 hpf and sections were labeled for pSmad3 (red) and nuclei (Dapi, blue). In (**C**,**D**), treatment was until 76 hpf and sections were labeled for pERK1/2 (red) and nuclei (Dapi, blue). Asterisk in (**A**) marks the area of the cell mass where pSmad3 labeling is evident. Arrowheads in (**B**,**D**) point at the ALE. White line in (**C**) marks the periphery of the cell mass. L, lens. Scale bars are 20 µm.

First, we treated mutant embryos from 26 hpf to 4 dpf and labeled eye tissue sections for pSmad3. We chose 4 dpf to assess the effect on TGFβ signaling since at this time point pSmad3 was consistently and clearly upregulated. We found that 4-PBA treatment blocked cell mass formation and no pSmad3-positive cells were present in the ALE (Figure 6A,B; *n* = 8 and 15, respectively). Some pSmad3-positive cells were present in adjacent cells, such as in the ciliary marginal zone (CMZ), as often observed in normal larvae (Figure 6B). 

Next, we treated embryos with 4-PBA from 26 hpf to 3 dpf and assayed for pERK1/2 labeling. Surprisingly, we found that pERK1/2 labeling was present even in treated larvae in which cell mass formation was blocked (Figure 6D; *n* = 14, *n* in C = 11).

These results indicate that EGFR activity in the ALE is not sufficient for driving cell mass formation and are consistent with a similar conclusion from previous work [31]. The results also suggest that 4-PBA does not inhibit TGFβ signaling at 1 dpf, since that would be expected to abolish EGFR activity as well.

### 3.7. Inhibition of mTOR Signaling Only Partially Limits Cell Mass Formation

**Figure 7:**
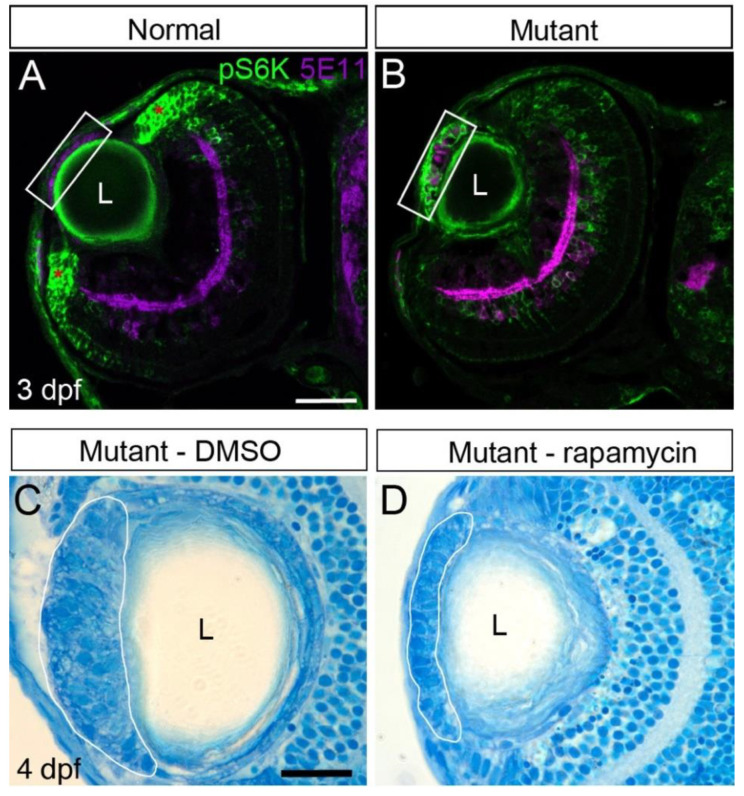
mTOR signaling contributes to cell mass growth. (**A**,**B**) Confocal single-plane sections from eyes of 3 dpf *plod3^vu222^* larvae labeled for pS6K (green) and with 5E11 antibody that marks nuclei of ALE cells as well as a subset of amacrine cells (purple). pS6K staining in the mutant is increased in the developing mass and reduced in the ciliary marginal zones (marked by asterisks in (**A**)). White rectangles mark the ALE region. Scale bar is 50 µm. (**C**,**D**) Histological sections from eyes of 4 dpf *plod3^vu222^* larvae treated from 26 hpf until ~96 hpf with vehicle (**C**) or rapamycin (**D**). White lines mark the periphery of cellular masses. Scale bar is 20 µm. L, lens.

Inhibition of EGFR signaling is aimed, at least in part, at blocking proliferation of LECs. Another pathway that is known to promote cell proliferation and growth is the mechanistic (formerly mammalian) target of rapamycin (mTOR) pathway. mTOR signaling can be activated by multiple factors, including TGFβ and EGFR signaling [32]. We therefore asked whether this pathway was upregulated in the ALE of *plod3* mutants as cell masses were forming. To test this possibility, we labeled tissue sections from eyes of 3 dpf normal and *plod3^vu222^* mutant larvae for the phosphorylated form of ribosomal protein S6 kinase (pS6K), which is a commonly used readout for activation of mTOR signaling [33,34]. We found that, whereas pS6K signal was missing from the ALE of normal larvae, it was strongly upregulated in forming cell masses in the mutants (Figure 7B; *n* = 8, *n* in A = 8).

mTOR signaling can be inhibited by rapamycin, which inhibits mTOR complex1 (mTORC1) activity [35], suggesting that treatment with rapamycin could block formation of lens cell masses in *plod3* mutants. We therefore treated embryos from 1 dpf until 4 dpf with different doses of rapamycin and evaluated the effect by gross morphology and by histological sections. From the different doses that were tested (2.5, 25 and 50 µM), treatment with 25 µM rapamycin led to partial inhibition of the lens phenotype, with treated larvae developing smaller masses than untreated ones (Figure 7C,D; *n* > 15 for each condition). However, the rescue achieved using this treatment was not as consistent as with inhibition of TGFβ or EGFR signaling or by treatment with 4-PBA. Therefore, while mTOR signaling likely contributes to the formation of cell masses, it is not essential for this pathology to develop.

## 4. Discussion

**Figure 8:**
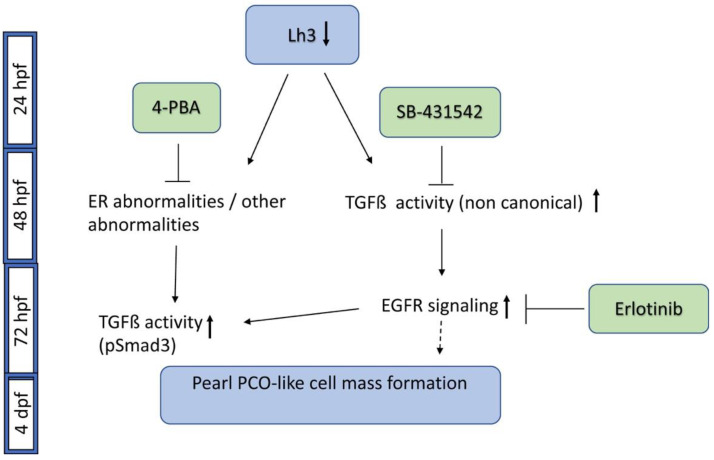
A schematic of pathways involved in plod3 mutant lens phenotype and small molecules that block phenotype development. Reduced function of Lh3 leads to ER abnormalities and possibly additional abnormalities by 2 dpf. Application of 4-PBA by ~28 hpf blocks phenotype development, likely by affecting these abnormalities. The TGFβ type I receptor inhibitor SB-431542 also blocks phenotype development if applied by ~28 hpf, suggesting early upregulation of non-canonical TGFβ signaling, as pSmad3 is not present before 3 dpf. Later on, around 72 hpf, increased EGFR signaling contributes to phenotype development downstream of TGFβ signaling, and blocking it by Erlotinib prevents phenotype development. Also around this time, as the cell mass develops, pSmad3-positive cells become apparent but the upregulation of this canonical TGFβ signaling occurs at a time point when blocking the pathway no longer prevents phenotype development. pSmad3 upregulation is dependent on the earlier phase of TGFβ signaling, on EGFR signaling and is also inhibited by 4-PBA. Timing of pathway and inhibitor activity as deduced from our experiments is depicted. The dashed line after EGFR signaling represents the finding that EGFR activation alone does not appear to drive cell mass formation.

In this work, we describe the identification of small molecules that can prevent formation of cellular masses from the ALE of *plod3* mutant zebrafish larvae. Together with our previous work [13], the findings further our knowledge of the mechanisms that underlie the development of the cell masses and show additional similarities to the pathogenesis of cataracts derived from lens epithelium in humans. A summary of the findings is shown in Figure 8.

### 4.1. Inhibition of Cell Mass Formation by 4-PBA

In our previous study, we showed that the lens cellular masses in *plod3*-deficient zebrafish larvae are derived from the ALE and proposed that their formation was driven in a non-cell-autonomous fashion by the abnormal lens capsule [13]. Nevertheless, the reduced function of Lh3 is expected to have cell-autonomous effects; Lh3 is a multifunctional enzyme with lysyl hydroxylase (LH), hydroxylysyl galactosyltransferase (GT) and galactosylhydroxylysyl glucosyltransferase (GGT) activities that functions in post-translational modifications of its substrates [36]. Hence, loss of Lh3 activity is expected to result in abnormalities in its substrate proteins leading to their misfolding and subsequent accumulation in the ER, thereby causing ER stress. Indeed, in Lh3 knockout mouse embryos, there was dilation of the ER with retention of Collagen IV, as well as enlargement of Golgi complexes and lysosomes [22,37]. Consistent with these reports, we also found altered appearance of the ER in the ALE and the adjacent developing corneal epithelium, which included mildly dilated ER. The expansion of the ER lumen in *plod3* mutants likely results from accumulation of misfolded proteins that were not properly modified by Lh3. This finding provided the basis for testing 4-PBA’s ability to rescue the lens phenotype. Interestingly, even though 4-PBA efficiently inhibited formation of lens cellular masses, our preliminary transcriptome analyses did not detect changes that are typical of ER stress in *plod3* mutants at 2 dpf, e.g., upregulation of *chop, bip* and splicing of *xbp1*. Nevertheless, we did detect increased transcription of chaperons and genes implicated in endoplasmic reticulum-associated protein degradation (ERAD) (Taler, Zatari and Inbal, unpublished). Both an increase in ER size and ERAD are known mechanisms through which cells cope with accumulation of misfolded proteins [38,39]. Hence, it is possible that 4-PBA indeed rescues the phenotype through its chaperon function by relieving potential stress caused by protein accumulation. Alternatively, it is also possible that the rescue is mediated via its function as an HDAC inhibitor. The exact mechanism needs further investigation. 

Our timing experiments show that 4-PBA activity is required to inhibit cell mass formation well before the phenotype becomes apparent. This result suggests that 4-PBA does not directly influence the mechanisms that drive the growth of LECs. Moreover, since the time window of 4-PBA activity parallels that of TGFβ, it raised the possibility that inhibiting TGFβ activity was the mechanism by which 4-PBA treatment yielded the rescue. However, this does not seem to be the case, as EGFR signaling, which is dependent on TGFβ activity, was not inhibited by 4-PBA treatment. Therefore, it appears that another mechanism promotes formation of cell masses in parallel to TGFβ and EGFR signaling.

The logic for testing 4-PBA as a candidate for prevention of LEC-derived cataract in *plod3*-mutant lenses is clear. However, an involvement of the ER in the pathogenesis of lens epithelium abnormalities could be unique to these mutants. It will be interesting to test whether 4-PBA can inhibit formation of PCO in models that are not related to Lh3 function. 

### 4.2. Inhibition of Cell Mass Formation by Blocking TGFβ and EGFR Signaling

We identified Erlotinib as an efficient inhibitor of lens cell mass formation in an unbiased small molecule screen. This identification is in strong agreement with previous studies that implicated EGFR signaling in PCO and identified EGFR-inhibiting drugs, including Erlotinib, Gefitinib and Lapatinib as candidates for use in prevention of PCO after cataract surgery [40,41,42,43,44,45,46]. Interestingly, a recent study suggested that only fibrotic PCO is prevented by EGFR inhibitors [46]. We, however, found that the lens phenotype of *plod3* mutants, which is more similar to pearl PCO, is efficiently prevented by Erlotinib. The reason for the different findings is unclear but might be because different experimental systems were used in the two studies.

Our analysis of pERK1/2 labeling suggested that EGFR activity was elevated just before the formation of lens cell masses. The relevance of this finding to the development of cell masses was confirmed by using Erlotinib during different time windows. Thus, EGFR signaling likely plays a more direct role in formation of lens cell masses. TGFβ signaling, on the other hand, is required for cell mass formation in *plod3* mutants much earlier, between 1 and 2 dpf [13], suggesting that it acts indirectly in promoting ALE cell masses. Furthermore, we showed that TGFβ signaling was required for the activation of EGFR signaling. These findings are consistent with data from a recent study which found that phosphorylation of EGFR occurred only 18 h after treatment of rat lens epithelial explants with TGFβ, suggesting that activation of EGFR by TGFβ is a downstream indirect signaling event [31]. 

TGFβ activity which drives cell mass formation appears to be from a non-canonical arm of the pathway, as pSmad3 is not upregulated by 2 dpf. Interestingly, in addition to its early role before 2 dpf, canonical TGFβ signaling is also upregulated in cellular masses at 3 dpf and onwards, as evidenced by positive pSmad3 labeling [13]. Whereas studies have shown that both canonical and non-canonical TGFβ signaling drive PCO formation (e.g., reviewed in [11,47]), in this study we found that the canonical activation of TGFβ signaling in the developing cell masses is not required for their development, as blocking it fails to rescue the phenotype. Rather, at 3 dpf, EGFR activity is required for cell mass formation and is dependent on earlier TGFβ signaling. Interestingly, the canonical TGFβ signaling at 3 dpf is dependent both on the earlier non-canonical signaling and on EGFR signaling [13], but its function is currently unclear.

EGFR activity appears to be permissive and not instructive, as evidenced by 4-PBA treatments that do not abolish pERK1/2 upregulation in the ALE but do inhibit cell mass formation. These observations are consistent with work showing that upregulation of EGFR signaling alone does not promote PCO [31].

Another point of interest is the apparent side effects of the different treatments. Whereas inhibition of TGFβ signaling often resulted in abnormal-looking ALECs [13], the histology of Erlotinib- and 4-PBA-treated larvae suggested that the ALECs of treated larvae were much more similar to normal ones. These data further support the notion that Erlotinib is a good candidate for the preventive treatment of PCO.

## 5. Conclusions

In conclusion, cell masses that develop in the lenses of zebrafish embryos and larvae with *plod3* loss of function show great similarity to pearl PCO. This similarity is demonstrated both by histology and by the underlying molecular mechanisms that drive the pathology formation, most prominently TGFβ and EGFR signaling. The non-canonical TGFβ signaling pathway plays a critical early and indirect role in the development of the pathology, whereas EGFR signaling plays a later, permissive role. Hence, in addition to providing information specifically relevant to the loss of Lh3 function, this work supports the notion that *plod3* zebrafish mutants can serve as an in vivo model for testing pharmacological strategies for prevention of PCO, the common complication of cataract surgeries.

## Data Availability

Not applicable.

## Supplementary Materials

The following supporting information can be downloaded at: https://www.mdpi.com/article/10.3390/cells12212540/s1, Figure S1: Lens phenotypes in live *plod3* mutant 4 dpf larvae; Figure S2: Section of a normal lens.
