# Supplementary material for: Identification of Small Molecules for Prevention of Lens Epithelium-Derived Cataract Using Zebrafish
Source: Cells. 2023 Oct 29;12(21):2540. doi: 10.3390/cells12212540 (PMC10650733; doi:10.3390/cells12212540)
Supplement: Supplementary file 1 [file cells-12-02540-s001.zip › cells-2599900-supplementary.pdf]

Supplementary information:

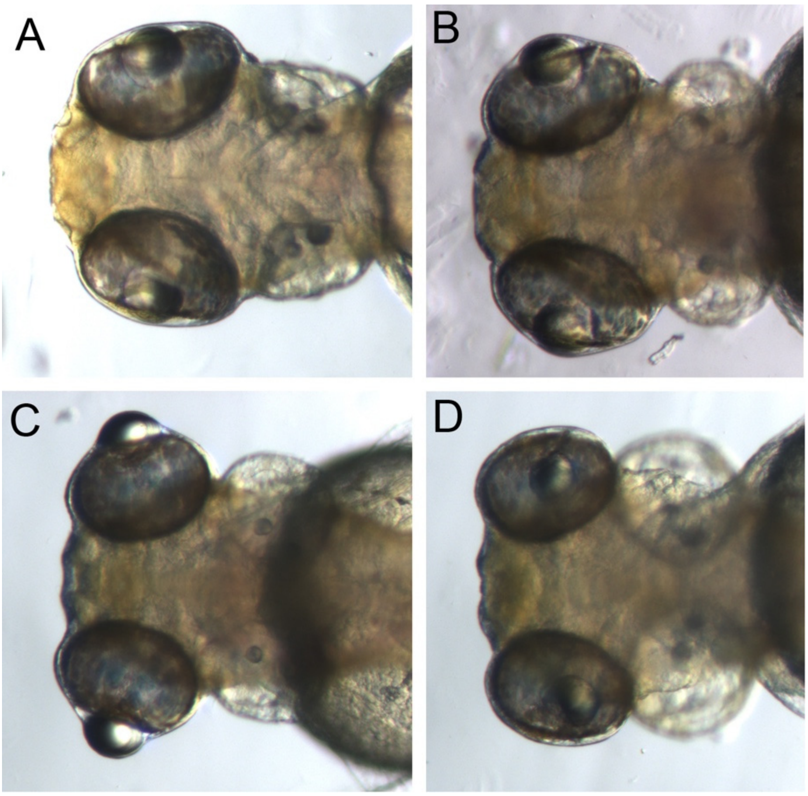

Supplementary Fig. S1: Lens phenotypes in live *plod3* mutant 4 dpf larvae.

(A) normal larva. (B) mutant larva, lens position classified as normal.

(C) mutant larva, lens position classified as outside. (D) mutant larva, lens position classified as inside.

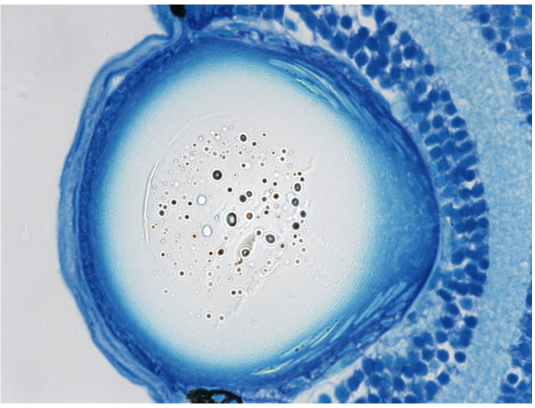

Supplementary Fig. S2: Section of a normal lens.

Histological section of a wild-type lens from a 4 dpf larva.
